# Syphilis – not just a STI

**DOI:** 10.1186/1757-1146-3-S1-P16

**Published:** 2010-12-20

**Authors:** Victoria Robinson

**Affiliations:** 1 Durham School of Podiatric Medicine, Durham, UK

## 

Syphilis has increased tenfold over the past decade, information for podiatrists that emphasizes it implications and relation to the lower limb is limited. The aim of the poster presentation is to draw attention to syphilis and is relevance within the 20^th^ century.

A literature review inspired the production of the poster. The purpose of the poster is to inform other podiatrists about the various signs, stages. symptoms and manifestations of syphilis. The poster covers many aspects which are relevant to the profession; it highlights the neurological, dermatological and wound presentations, as well as the subsequent management and diagnosis.

The poster has been designed to inform peers, with the aim to bring syphilis and its complications to the forefront with regards to the recent increase in the condition thus highlighting its relevance to the lower limb and podiatry.

